# *Elettaria cardamomum* (L.) Maton Essential Oil: An Interesting Source of Bioactive Specialized Metabolites as Inhibitors of Acetylcholinesterase and Butyrylcholinesterase

**DOI:** 10.3390/plants12193463

**Published:** 2023-10-02

**Authors:** Marta Pavarino, Arianna Marengo, Cecilia Cagliero, Carlo Bicchi, Patrizia Rubiolo, Barbara Sgorbini

**Affiliations:** Dipartimento di Scienza e Tecnologia del Farmaco, Università di Torino, Via Pietro Giuria 9, I-10125 Turin, Italy; m.pavarino@unito.it (M.P.); arianna.marengo@unito.it (A.M.); cecilia.cagliero@unito.it (C.C.); carlo.bicchi@unito.it (C.B.)

**Keywords:** *Elettaria cardamomum* essential oil, acetylcholinesterase, butyrylcholinesterase, bio-guided fractionation

## Abstract

*Elettaria cardamomum* (L.) Maton (Zingiberaceae family) is a plant traditionally used in Ayurvedic and Chinese medicine. In this work, the essential oil of *E. cardamomum* was found to inhibit the enzymes AChE (62.6% of inhibition, IC_50_ 24.9 μg/mL) and BChE (55.8% of inhibition, IC_50_ 25.9 μg/mL) by performing an in vitro colorimetric assay using the Ellman method. A bio-guided fractionation approach was used to isolate fractions/pure compounds that were tested individually to evaluate their activity. The resulting oxygenated fraction was found to be active against both AChE (percentage inhibition 42.8%) and BChE (percentage inhibition 63.7%), while the hydrocarbon fraction was inactive. The activity was attributed to a pool of oxygenated terpenes (α-terpinyl acetate, 1,8-cineole, linalool, linalyl acetate, and α-terpineol) that synergistically contributed to the overall activity of the essential oil.

## 1. Introduction

Alzheimer’s disease (AD) is a progressive neurodegenerative disease that is considered to be a major cause of dementia due to the increasing ageing of the population worldwide. It is a disease that severely affects patients’ daily activities, which has a significant impact on society (i.e., families, communities, and healthcare systems) [1,2]. According to estimates from WHO, more than 55 million people are living with dementia, and this number is expected to triple worldwide by 2050 [3,4]. The etiology of cognitive and memory impairment is still not fully understood. What is known so far about the pathogenesis of this disease includes several different causes leading to neurodegeneration, which follow each other and are closely linked. The causes are thought to be the deposition of extracellular amyloid plaques and the intracellular alteration of neurofibrillary tangles (NFTs), which probably lead to the deterioration of synapses and the death of neurons in parallel pathways [5]. The therapies approved by the FDA (Food and Drug Administration) and the EMA (European Medicinal Agency) act at the synaptic level, including acetylcholinesterase (AChE) inhibitors (i.e., donepezil, rivastigmine, and galanthamine) and the N-methyl-D-aspartate receptor antagonist memantine. Recently, two anti-amyloid beta antibodies, lecanemab and aducanumab, were approved by the FDA (2021 and 2023, respectively) under an accelerated approval process based on the drugseffect on a surrogate endpoint, although their clinical benefit is not yet certain, so they are still at a preliminary stage [6,7]. Today, AChE inhibitors are the only class of drugs specifically used to treat AD and increase the ACh levels in synapses. This treatment is time-limited, as AChE levels decrease in the advanced stages of the disease. As butyrylcholinesterase (BChE) levels increase significantly and acetylcholine levels decrease dramatically in the late stage of AD, it is also important to find effective BChE inhibitors that increase the acetylcholine levels in the advanced stages of AD. These observations have suggested a possible improvement in the therapy of AD with acetyl and butyrylcholinesterase inhibitors, which represent an interesting new line of research for drugs against AD [8,9].

Essential oils are complex mixtures of volatile compounds obtained from a single plant species via steam distillation, dry distillation, or mechanical processes [10]. They are best known for their use in flavours, fragrances, and cosmetics, but have traditionally been used for many other purposes, including religious ceremonies and as therapeutic agents [11]. Their traditional use has led to increasing attention being paid to their biological activity, including their activity at the level of the central nervous system. Numerous studies can be found in the literature, but they often focus only on the inhibition of AChE. When the inhibition of both enzymes is investigated, many essential oils turn out to be selective inhibitors of one of the enzymes [12,13]. In this context, it becomes clear how important it is to find new agents that can inhibit both cholinesterase enzymes.

*Elettaria cardamomum* (L.) Maton is an herbaceous perennial plant (family: Zingiberaceae) native to southern India and Sri Lanka, but it is also naturalised in Guatemala and Tanzania and cultivated worldwide (e.g., in Nepal, Indonesia, Honduras, Vietnam, Cambodia, and Malaysia). This plant has been used for thousands of years by ancient civilisations (e.g., the Indians, Greeks, and Romans) for culinary purposes and its healing properties in traditional medicine, making it one of the oldest spices used [14]. Its traditional medicinal uses include asthma, gingivitis, digestive, kidney and heart diseases, and metabolic syndrome [15,16,17]. Nowadays, it is mainly used as a flavouring and fragrance, and is becoming increasingly popular.

The essential oil of *E. cardamomum* has already been studied for its antioxidant, antibacterial, antifungal, antispasmodic, antidiarrheal, and anticarcinogenic properties [18,19,20].

In contrast, to the best of the authors’ knowledge, there is only one paper in the literature that has reported the evaluation of cardamom essential oil as an inhibitor of AChE and BChE [21], but the study refers to an essential oil whose chemical composition does not match that defined in the standard ISO 4733-2004 [22]. Of the components of the essential oil with a standard composition, only terpinyl acetate was studied [23], without evaluating the other possible compounds that could act as additional/synergic co-inhibitors.

In this context, this study focuses on evaluating the inhibitory effect of different samples of cardamom essential oil on both AChE and BChE by applying a bio-guided fractionation approach to identify, for the first time, the compounds responsible for this inhibitory effect. In addition, the stability of the chemical composition and inhibitory activity is evaluated as a function of different storage conditions.

## 2. Results and Discussion

### 2.1. Chemical Composition of the Investigated Essential Oils

In the present study, six batches of commercial samples of cardamom essential oil (EO), obtained via steam distillation from fruits of botanically authenticated plants from Guatemala, were investigated. In addition, a sample of whole cardamom fruits (opened capsules and seeds) purchased from a local herbal specialty shop (geographical origin Guatemala) was hydrodistilled for 4 h in Clevenger apparatus and compared with the samples obtained via steam distillation. 

**Figure 1:**
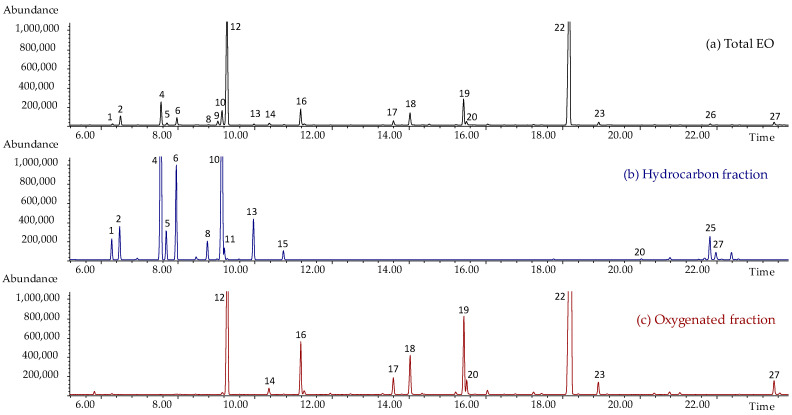
GC-MS profile of (**a**) total cardamom EO (batch 5) of (**b**) hydrocarbon and (**c**) oxygenated fractions analysed in a conventional apolar GC column. Peak numbers refer to Table 1.

The above EOs were analysed using gas chromatography (GC) coupled with mass spectrometry (MS) to determine their composition and characterise all the potential constituents of cardamom EO that contribute to the biological activity studied. The normalised relative percentage abundances (calculated from the absolute areas normalised to the internal standard nonane using response factors [24,25]) of all the detected compounds were determined and used to compare the EO compositions. Figure 1a shows the GC-MS profile of one of the batches of cardamom EO analysed in a conventional apolar GC column. 

Table 1 shows the average chemical composition of the analysed batches of cardamom EOs and the chemical composition of the hydrodistilled EO. The chemical compositions of all the batches analysed are reported in the supplementary materials (Table S1).

The compositions of all the EO batches agree with the information in ISO 4733:2004 and the literature [14,22], which confirms the authenticity of the samples analysed. As Table 1 shows, the cardamom EO is characterised by a preponderance of oxygenated compounds: 1,8-cineole and terpinyl acetate are the most abundant, with more than 30% and 40%, respectively. The EO obtained via hydrodistillation also has a composition that is mostly the same as that of the ISO standard (with the exception of 4-terpineol, α-terpineol, and linalyl acetate, whose percentage abundances are lower than those of the ISO standard). The differences observed in the EO obtained via hydrodistillation were due to the fact that the raw plant material was immersed in water during the distillation. Indeed, compounds that are more soluble in water tend to remain in water [26], which leads to a reduction in percentage in the obtained essential oil.

### 2.2. In Vitro Inhibitory Activity of the Investigated Essential Oil against Acetylcholinesterase and Butyrylcholinesterase

The inhibitory activity of the *E. cardamomum* EO against AChE and BChE was tested using a spectrophotometric assay following the protocol of Rhee [27], which was optimised by (1) using DMSO as the solvent for the EO dilution and adding TWEEN20 as a surfactant to buffer B to increase the solubility of the essential oils, after confirming that they did not negatively affect the enzymatic activity, and (2) lowering the substrate concentration to be within the linearity range of the enzyme (see Section 3.2 for details). Table 2 shows the percentage inhibitions of AChE and BChE obtained by testing all the cardamom EOs at a concentration of 38 µg/mL (concentration in the final reaction mixture). Galanthamine was used as a positive control and tested at a concentration of 1 µg/mL (concentration in the final reaction mixture).

Cardamom EO has proven promising in the treatment of both early and advanced AD thanks to its dual inhibitory effect on AChE and BChE. Indeed, all the batches effectively inhibited AChE and BChE at the concentration studied. 

All the samples showed comparable activity against AChE, as confirmed by a one-way analysis ANOVA (*p* values > 0.05). One of the compounds responsible for this activity was the main compound 1,8-cineole, which is known to actively inhibit AChE, although the mechanism of this is still unclear [28]. α-Terpinyl acetate is the other main EO compound and is characterised by a competitive inhibition of the enzyme AChE and mixed inhibition of BChE [23]. Since all the EOs tested had similar contents of 1,8-cineole and α-terpinyl acetate, it is not surprising that a similar inhibitory effect was observed.

The inhibitory concentration that halved the enzyme activity under the experimental conditions considered (IC_50_) was determined to better compare the inhibitory activities for all the commercial batches, the hydrodistilled EO, galanthamine, and the two main compounds (i.e., 1,8-cineole and α-terpinyl acetate). Table 3 shows the IC_50_ values for all the samples tested. The IC_50_ values confirmed that all the samples effectively inhibited AChE and BChE and exhibited inhibitory activity that was, on average, 40-fold lower than that of galanthamine over AChE and 10-fold lower than that of galanthamine over BChE, with the exception of 1,8-cineole, which was found to be an inactive inhibitor of BChE. The IC_50_ value of α-terpinyl acetate for AChE was not calculated, because 50% inhibition was never achieved under the adopted experimental conditions.

### 2.3. Identification of Bioactive Components by Bioguided Assay Fractionation

*E. cardamom* EO (batch 5) was subjected to bioguided assay fractionation due to its promising cholinesterase inhibitory activity. The oxygenated and hydrocarbon fractions of the investigated EO were isolated using automated flash chromatography and individually subjected to acetylcholinesterase and butyrylcholinesterase inhibition assays.

The *E. cardamomum* EO was subjected to flash chromatography separation on pre-packed 50 µm silica gel cartridges using a gradient of 0–30% ethyl acetate in petroleum ether. The total fractionation time was 20 min. The hydrocarbon fraction was completely eluted in the first isocratic step using 100% petroleum ether. The oxygenated fraction was eluted in the second gradient step when the ethyl acetate increased from 0% to 30%. The amount of EO to be fractionated was 900 mg ± 15mg and the yields of the oxygenated and hydrocarbon fractions were 675.7 mg ± 7.3 mg and 17 mg ± 2.5 mg, respectively, which were the averages of three fractionations. Each isolated fraction was analysed using GC-MS to determine the chemical composition of the fraction. Figure 1b,c show, respectively, the GC-MS chromatograms of the hydrocarbon and oxygenated fractions obtained via the fractionation of the cardamom EO, and Table 1 shows their relative chemical compositions.

Both the hydrocarbon and oxygenated fractions were subjected to AChE and BChE inhibition tests at the concentrations in which they are present in the EO. Only the oxygenated fraction inhibited AChE by 42.8% ± 0.5 and BChE by 63.7% ± 3.0, as shown in Table 4, while the hydrocarbon fractions were completely inactive in the inhibition tests. It can also be seen that the inhibitory activity of the oxygenated fraction was lower for AChE and higher for BChE compared to the average activity of all the commercial samples.

To further investigate the activity of the oxygenated fraction, all the compounds in this fraction with a percentage area greater than 1% of the total EO were tested for both enzymes (at the concentration within the EO) to see if they had an effect on the overall EO inhibitory activity. The oxygenated fraction consisted mainly of 1,8-cineole (28.5%) and terpinyl acetate (52.1%). These compounds were tested as a pure standard at the same concentration as found in the EO and the results showed that they inhibited AChE by 55.4% ± 0.7 and 17.0% ± 1.0, respectively, while BChE was inhibited by 39% ± 2.7 by α-terpinyl acetate; 1,8-cineole had no activity on this enzyme (see Table 4). In AChE, the inhibitory activity of the mixture of α-terpinyl acetate and 1,8-cineole (each at the concentration within the EO) was less than the sum of the activities of the individual compounds, whereas, in the case of butyrylcholinesterase, 1,8-cineole, which is not active alone, enhanced the inhibition of the enzyme when tested together with α-terpinyl acetate. The other compounds in the oxygenated fraction with a percentage area greater than 1% (linalool, α-terpineol, and linalyl acetate) were then tested individually to determine whether they contributed to the overall activity EO together with 1,8-cineole and α-terpinyl acetate. Only linalool and linalyl acetate were found to be active in inhibiting BChE when tested individually, as shown in Table 4. Therefore, these two compounds were tested together with 1,8-cineole and α-terpinyl acetate, resulting in inhibitory activity against both enzymes comparable to that of the total essential oil. Finally, α-terpineol, which was shown to be inactive against both enzymes when tested individually, was also tested together with the other compounds in the oxygenated fraction, which did not further increase the inhibitory activity of the mixture for either enzyme.

**Figure 2:**
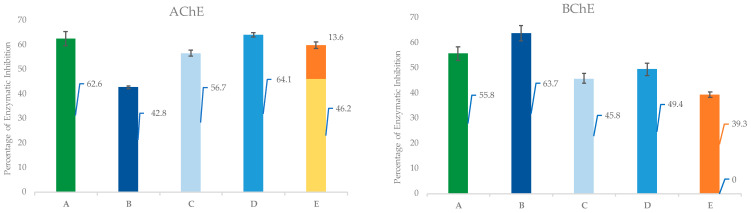
Comparison of experimentally measured percentage inhibitions for the total EO, its fractions, and mixtures of bioactive compounds with the expected inhibition of 1,8-cineole and α-terpinyl acetate as the only bioactive compounds in the essential oil, extrapolated from the IC_50_ dose–response curve. Legend: *A*: EO batches average experimental inhibitory activity; *B*: EO oxygenated fraction experimental inhibitory activity; *C*: 1,8-cineole and α-terpinyl acetate mixture experimental inhibitory activity; *D*: mixture of 1,8 cineole, α-terpinyl acetate, linalool, linalyl acetate, and α-terpineol experimental inhibitory activity; and *E*: 1,8-cineole (displayed in yellow) and α-terpinyl acetate (displayed in orange) expected inhibitory activity extrapolated from IC_50_ dose–response curve.

In the case of AChE, the mixture of 1,8-cineole and α-terpinyl acetate had a higher inhibitory activity than the oxygenated fraction, which was further enhanced when tested together with three other minority components of the oxygenated fraction (linalool, linalyl acetate, and alpha-terpineol), resulting in the same inhibition as the whole EO (Figure 2). This suggests that other compounds were present in the oxygenated fraction tested that antagonised this synergistic effect between these five compounds.

In contrast, the mixture of the two main compounds for BChE had a lower activity than the oxygenated fraction, which was increased by the union of the three minority compounds, but without balancing them. In this case, this led to the consideration that there may be other compounds (at very low concentrations or in trace amounts) that further increase the biological activity of these five compounds.

### 2.4. Effect of Light and Temperature Exposure on EO’s Biological Activity

The stability of the cardamom EO was investigated both in terms of its chemical composition and inhibitory activity. For this purpose, different aliquots of the cardamom EO (batch 6) were kept for eight months under three different storage conditions, i.e., in the dark in an amber vial at room temperature (standard conditions), in the light in a transparent vial at room temperature, and in the dark in an amber vial placed at regular intervals twice a week for 60 min at a temperature of 60 °C. All the EO aliquots were opened for five seconds each day to simulate daily consumer use.

**Figure 3:**
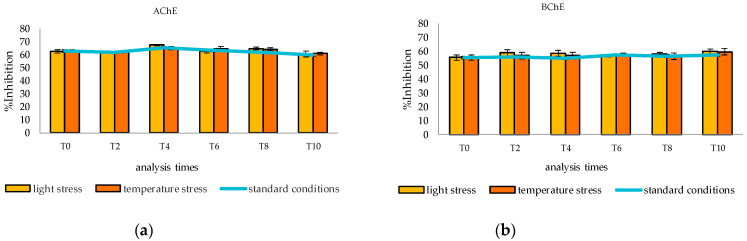
Inhibitory activity against AChE (**a**) and BChE (**b**) of cardamom EO stored under standard conditions (darkness and room temperature), under light and temperature stresses.

Both the inhibitory activity against AChE and BChE and chemical composition were evaluated. The chemical composition was assessed using GC-MS analyses before the start of the storage monitoring (t_0_), then every two weeks for the first two months (t_1_, t_2_, t_3_, and t_4_), and every month for the last six months (t_5_, t_6_, t_7_, t_8_, t_9_, and t_10_). The inhibitory activity was tested once a month (t_0_, t_2_, t_4_, t_6_, t_8_, and t_10_). As Figure 3 shows, no significant differences were found in the inhibitory activity of the cardamom EO under the stress conditions (light and/or temperature) compared to the standard conditions. This was due to the fact that the inhibitory activity is due to the oxygenated compounds, which are poorly affected by both light and temperature. A one-way ANOVA analysis was performed and the results obtained confirmed that there were no significant differences (*p*-values > 0.05). 

As expected from the trend in the bioactivity, the chemical composition of the cardamom EO did not change significantly under the storage conditions studied (data not shown). Figure S1 shows the GC-MS profiles of the cardamom EO at t_0_ and t_10_, respectively, under standard conditions, under light, and under temperature stress.

## 3. Materials and Methods

### 3.1. Reagent

Dimethyl sulfoxide (DMSO), acetylcholinesterase (AChE) from *Electrophorus electricus* and butyrylcholinesterase (BChE) from equine serum, acetylthiocholine iodide (ATCI), butyrylthiocholine iodide (BTCI), 5,5′-dithiobis-2-nitrobenzoic acid (DTNB), galanthamine hydrobromide, and all the buffer components (TWEEN20, bovine serum albumin, Trizma base, Trizma HCl, MgCl_2_·6H_2_O), cyclohexane, nonane, petroleum ether, ethyl acetate, 1,8-cineole, linalool, linalyl acetate, 4-terpineol, and α-terpineol were purchased from Merck Life Science S.r.l. (Milan, Italy).

Six batches of *Elettaria cardamomum* (L.) Maton EO were obtained via steam distillation from botanically identified plant material (dried fruits, geographical origin Guatemala) and supplied by Erboristeria Magentina (Poirino, Torino, Italy). The hydrodistilled EO was obtained by submitting 100 g of dried fruits (same geographical origin) purchased from a local herbal shop to hydrodistillation for 4 h in Clevenger apparatus (a pic of the plant used to obtain the hydrodistilled EO is reported in the Supplementary Material Figure S2). The EO was collected and stored in the dark at +4 °C until use.

### 3.2. In Vitro Acetylcholinesterase and Butyrylcholinesterase Inhibition Test

The protocol used in this study was that of Rhee et al., with slight modifications [26]. The following buffers were used: buffer A (50 mM Tris-HCl pH 8), buffer B (50 mM Tris-HCl pH 8, with the addition of 0.1% of bovine albumin and 0.1% of TWEEN20), and buffer C (50 mM Tris-HCl pH 8, with the addition of 0.1 M of NaCl and 0.02 M of MgCl_2_ 6H_2_O). The enzyme solution of 0.22 U/mL was prepared in buffer B. The acetylthiocholine iodide (ATCI) solution of 1.5 mM was prepared in Millipore water, while the butyrylthiocholine iodide (BTCI) solution was prepared in buffer A. Ellman’s reagent solution at 3 mM was prepared in buffer C. Essential oil stock solutions at 15mg/mL and a galanthamine solution at 0.4 mg/mL, used as positive controls, were prepared in dimethyl sulfoxide (DMSO).

The reagents were added to the cuvette in the following order: 150 µL of the ATCI or BTCI solution, 750 µL of the Ellman’s reagent solution, 900 µL of buffer B, 5 µL of the essential oil/galanthamine solution, and, finally, 150 µL of the enzyme solution. The reaction mixture was incubated at 30 °C for 6 min. The absorbance values were measured at 405 nm after incubation. The control solution, in which the enzyme was fully active, was prepared by replacing the EOs/galanthamine solution with 5 µL of pure DMSO. The control and sample blank solutions were prepared by replacing 150 µL of the enzyme solution with 150 µL of buffer A.

The percentage of the AChE or BChE inhibition was measured according to the equation below:

% Inhibition = ΔA (Control) − ΔA (Sample)/ΔA (Control) × 100

ΔA (Control) = A_412_ (Control) − A_412_ (Control Blank)

ΔA(Sample) = A_412_ (Sample) − A_412_ (Sample Blank)

The concentration–response curve was determined for each inhibitor by plotting the inhibitory activity against AChE or BChE as a function of the inhibitor concentration in the reaction mixture.

### 3.3. Flash Column Chromatography

The fractionation was carried out using a system for flash column chromatography PuriFlash 450 (Sepachrom, Rho, Milano, Italy), equipped with both UV and Evaporative Light Scattering (ELSD) detectors. The solvents used for the mobile phase were petroleum ether (solvent A) and ethyl acetate (solvent B); a linear elution gradient from 100% A to 70% A and 30% B in 20 min was used. Pre-packed silica Sphera 50 µm silica cartridges (Sepachrom, Rho, Milano, Italy) were used. The eluent flow was kept at 25 mL/min and the amount of EO fractionated was 900.0 mg.

### 3.4. GC-MS Analysis Conditions

The EO solutions and those of their respective fractions were prepared in cyclohexane at a concentration of 5.0 mg/mL, with 5 μL of nonane solution in cyclohexane (20 mg/mL) as internal standard, and analysed using GC-MS.

The GC-MS analyses were carried out on an Agilent 6890 GC unit coupled to an Agilent 5975 MSD (Agilent, Little Falls, DE, USA) equipped with a MPS-2 multipurpose sampler (Gerstel, Mülheim a/d Ruhr, Germany). For the data elaboration, an Agilent Chemstation (release F.01.03.2357) was used.

GC conditions: injector temperature: 250 °C; injection mode: split; ratio: 1/20; carrier gas: helium; constant flow rate: 1 mL/min; and columns: MEGA5 (95% polydimethylsiloxane, 5% phenyl) 30 m × 0.25 µm *d_f_*, 0.25 mm *d_c_*. Temperature program: 50 °C (1 min)//3 °C/min//250 °C//250 °C (5 min). MSD conditions: MS operated in EI mode (70 eV); scan range: from 35 to 350 amu; dwell time: 34 ms; ion source temperature: 250 °C; quadrupole temperature: 150 °C; and transfer line temperature: 270 °C. The EO compounds were identified by comparing both their linear retention indices (*I^T^*s), calculated with a C9–C25 hydrocarbon mixture and their mass spectra, either against those of the authentic samples and/or from commercially available mass spectral libraries [29].

### 3.5. Storage Conditions

The EO of cardamom (batch 6) was divided into three aliquots. The first aliquot was kept in a transparent vial and continuously exposed to light. The second aliquot was kept in the dark in a closed amber vial to mimic the conditions normally prescribed to consumers (standard conditions). The third aliquot was always kept in the dark in a closed amber vial, but was exposed to thermal stress at 60 °C for 60 min twice a week. All the aliquots were opened for five seconds each day to simulate daily consumer use. The samples were analysed at baseline (t_0_) and as follows. The chemical composition was assessed using a GC-MS analysis before the start of the storage monitoring (t_0_), then every two weeks for the first two months (t_1_, t_2_, t_3_, and t_4_), and every month for the last six months (t_5_, t_6_, t_7_, t_8_, t_9_, and t_10_). The inhibitory effect was tested once a month (t_0_, t_2_, t_4_, t_6_, t_8_, and t_10_).

### 3.6. Anova Test and IC_50_ Calculation

The Microsoft Excel programme was used for the Anova test. For the IC_50_ calculation, an online programme from AAT Bioquest was used (https://www.aatbio.com/tools/ic50-calculator, accessed on 10 May 2023).

## 4. Conclusions

In this study, a bioguided fractionation approach was used to investigate, in detail, the inhibitory activity of cardamom essential oil. This involved searching for bioactive volatile compounds that inhibit both AChE and BChE enzymes.

The results showed that cardamom essential oil is a promising source of compounds that inhibit both cholinesterase enzymes. This was confirmed for different batches of EO, even under different storage conditions. The bioactive compounds were those present in the oxygenated fraction, and the most active compounds were 1,8-cineole for AChE and α-terpinyl acetate for BChE, respectively. Moreover, the inhibitory activity was the result of the simultaneous action of several compounds, suggesting an additive/synergetic effect of the compounds in combination.

In view of the above considerations, cardamom essential oil is very promising for further in-depth studies in search of a complementary treatment to conventional therapy.

## Figures and Tables

**Table 1:** Chemical composition expressed as relative percentage abundance (% area) of the batches analysed (average) and of the essential oil obtained via hydrodistillation (Hydrodist EO) together with the composition of hydrocarbon and oxygenated fraction obtained via fractionation of batch 5 EO. Analytical percent relative standard deviation (RSD%) <3%. Legend: HYDR, hydrocarbon fraction; OXY, oxygenated fraction; tr: trace; and n.d.: not detected.

#	Exp*I*^t^_s_	Lit*I*^t^_s_	Compounds	Batches (Average ± σ)	Hydrodist OE	HYDR	OXY	ISO 4733:2004 Min (Central America/ Guatemala)	ISO 4733:2004 Max (Central America/ Guatemala)
1	931	931	α-thujene	0.2 ± 0.03	0.2	2.3			
2	939	939	α-pinene	1.4 ± 0.1	1.0	3.8		1.0	2.0
3	956	953	camphene	tr		0.2			
4	978	976	sabinene	3.4 ± 0.6	1.9	36.8		3.0	5.0
5	983	980	β-pinene	0.3 ± 0.05	0.3	3.3			
6	992	991	β-myrcene	1.3 ± 0.4	1.3	11.1		tr	2.5
7	1010	1005	α-phellandrene	tr		0.4			
8	1022	1018	α-terpinene	0.1 ± 0.02	0.4	2.2			
9	1032	1029	*p*-cymene	0.5 ± 0.2	0.2	tr			
10	1036	1031	limonene	2.2 ± 0.2	2.4	26.4		2.0	3.0
11	1038	1031	β-phellandrene	n.d.		1.2			
12	1041	1038	1,8-cineole	30.5 ± 0.7	33.6		28.5	27.0	35.0
13	1065	1062	γ-terpinene	0.2 ± 0.1	0.7	5.0			
14	1078	1075	*cis*-sabinene hydrate	0.4 ± 0.1	tr		0.5		
15	1089	1088	α-terpinolene	tr	0.4	1.1			
16	1104	1103	linalool	3.4 ± 0.2	5.0		4.2	3.0	6.0
17	1188	1184	4-terpineol	0.8 ± 0.04	2.5		1.4	0.8	1.5
18	1202	1198	α-terpineol	1.9 ± 0.3	4.1		3.4	tr	2.5
19	1254	1251	linalyl acetate	6.1 ± 0.7	1.7		6.6	4.0	6.0
20	1257	1255	geraniol	0.8 ± 0.1	1.7		1.1		
21	1276	1270	geranial	0.3 ± 0.03	0.2				
22	1355	1350	α-terpinyl acetate	43.9 ± 0.3	40.3		52.1	35.0	45.0
23	1382	1383	geranyl acetate	0.7 ± 0.1	0.7		1.0		
24	1426	1418	trans-β-caryophyllene	n.d.	n.d.	0.1			
25	1486	1485	β-selinene	0.2 ± 0.1	n.d.	3.3			
26	1490	1494	α-selinene	n.d.	n.d.	0.3			
27	1566	1565	(*Z*)-nerolidol	0.8 ± 0.1	0.5		1.2	0.5	1.0
Total hydrocarbon compounds				9.9 ± 1.2					
Total oxygenated compounds				89.5 ± 1.4					

**Table 2:** Percentage inhibition of AChE and BChE by *E. cardamomum* EO samples compared to galanthamine as positive control. Values represent the average of three tests. σ: Standard deviation values.

Sample	AChE Inhibition (%)	BChE Inhibition (%)
Galanthamine	68.4 ± 2.9	24.7 ± 0.6
Batch 1	59.2 ± 5.6	57.2 ± 0.5
Batch 2	60.5 ± 3.1	59.4 ± 1.3
Batch 3	66.6 ± 1.9	54.4 ± 0.9
Batch 4	64.0 ± 0.3	55.4 ± 1.6
Batch 5	62.4 ± 1.1	52.4 ± 2.0
Batch 6	63.5 ± 1.5	61.6 ± 1.2
Hydrodistilled EO	62.2 ± 2.1	57.3 ± 2.0

**Table 3:** IC_50_ values of tested EO samples (on commercial batch and hydrodistilled sample) and of the two main bioactive components together with their standard deviation value (*n* = 3).

Inhibitor	IC_50_ AChE (μg/mL)	IC_50_ BChE (μg/mL)
Galanthamine	0.468 ± 0.002	3.40 ± 0.0225
1,8-Cineole	14.1 ± 0.556	not active
α-Terpinyl acetate	Activity lower than 50%	21.9 ± 0.623
Commercial batch	24.9 ± 0.350	25.9 ± 1.71
Hydrodistilled EO	24.3 ± 0.248	37.2 ± 3.24

**Table 4:** Inhibitory activity against AChE and BChE of the hydrocarbon fraction, the oxygenated fraction, and its compounds at the concentrations in which they are present in EO. * The values represent the average of three assays; n.a.: not active.

Sample	AChE Inhibition (%) *	σ	BChE Inhibition (%) *	σ
Cardamom EO commercial sample mean	62.6	2.9	55.8	2.7
Oxygenated fraction	42.8	0.5	63.7	3.0
Hydrocarbon fraction	n.a.		n.a.	
α-terpinyl acetate	17.0	1.0	39.0	2.7
1,8-cineole	55.4	0.7	n.a.	
Mixture of α-terpinyl acetate and 1,8 cineole	56.7	1.2	45.8	2.0
Linalool	n.a.		5.0	1.4
α-terpineol	n.a.		n.a.	
Linalyl acetate	n.a.		10.3	3.3
Mixture of 1,8 cineole, α-terpinyl acetate, linalool, and linalyl acetate	63.8	1.0	50.3	2.2
Mixture of 1,8 cineole, α-terpinyl acetate, linalool, linalyl acetate, and α-terpineol	64.1	2.1	49.4	2.5

## Data Availability

The data presented in this study are available on request from the corresponding authors.

## Supplementary Materials

The following supporting information can be downloaded at: https://www.mdpi.com/article/10.3390/plants12193463/s1, Table S1. Chemical composition expressed as relative percent abundance (% Area) of the commercial batches analysed. Analytical percent relative standard deviation (RSD%) < 3%.; Figure S1: GC-MS patterns of the cardamom EO at t_0_ and at t_10_, respectively under standard conditions, under light and under temperature stress.; Figure S2. Dried fruits of cardamom used to obtain hydrodistilled EO.
