# Supplementary material for: Elettaria cardamomum (L.) Maton Essential Oil: An Interesting Source of Bioactive Specialized Metabolites as Inhibitors of Acetylcholinesterase and Butyrylcholinesterase
Source: Plants (Basel). 2023 Oct 2;12(19):3463. doi: 10.3390/plants12193463 (PMC10575282; doi:10.3390/plants12193463)
Supplement: Supplementary file 1 [file plants-12-03463-s001.zip › plants-2624902-supplementary.pdf]

Supplementary materials

Table S1. Chemical composition expressed as relative percent abundance (% Area) of the commercial batches analysed. Analytical percent relative standard deviation (RSD%) < 3%.

**Compounds**	**ExpI^T**	**LitI^T**	**Batch 1**	**Batch 2**	**Batch 3**	**Batch 4**	**Batch 5**	**Batch 6**
α-thujene	929	931	0.2	0.1	0.2	0.2	0.2	0.2
α-pinene	937	939	1.2	1.5	1.5	1.5	1.4	1.4
sabinene	976	976	2.6	2.6	3.7	3.7	3.9	4.0
β-pinene	980	980	0.3	0.4	0.3	0.3	0.3	0.4
β-myrcene	991	991	0.7	1.2	1.1	1.3	1.4	1.9
α-terpinene	1020	1018	0.1	0.1	0.1	0.1	0.1	0.1
p-cymene	1029	1026	0.5	0.4	0.3	0.6	0.9	0.3
limonene	1033	1031	1.9	2.4	2.1	2.3	2.4	2.3
1,8-cineole	1038	1033	29.9	30.9	30.2	31.3	30.9	29.7
γ-terpinene	1062	1062	0.2	0.2	0.2	0.2	0.1	0.3
cis-sabinene hydrate	1075	1068	0.5	0.3	0.5	0.4	0.4	0.4
linalool	1103	1098	3.4	3.4	3.5	3.2	3.3	3.8
4-terpineol	1184	1177	0.9	0.8	0.8	0.8	0.8	0.9
α-terpineol	1198	1189	1.8	1.5	1.9	2.3	1.7	2.1
linalyl acetate	1254	1251	6.5	7.2	5.9	5.5	5.4	6.0
geraniol	1256	1255	0.7	0.7	0.8	0.8	0.7	0.9
geranial	1274	1270	0.2	0.3	0.3	0.2	0.3	0.3
α-terpinyl acetate	1353	1350	46.8	43.1	44.4	43.0	43.5	42.3
geranyl acetate	1383	1383	0.6	0.7	0.5	0.6	0.7	0.7
β-selinene	1489	1485	0.3	0.3	0.2	0.3	0.2	0.2
(*Z*)-nerolidol	1565	1565	0.7	0.9	0.9	0.8	0.8	0.8

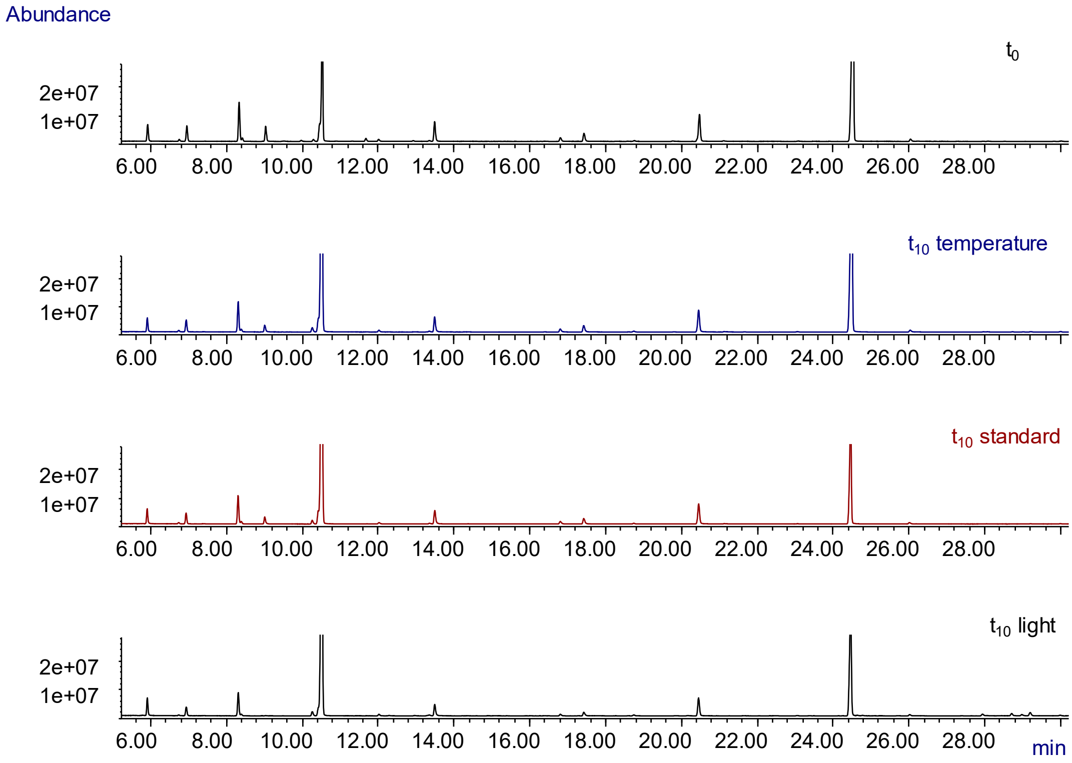

Figure S1. GC-MS profiles of the cardamom EO (batch 6) at t_0 and at t_{10}, respectively under standard conditions, under light and under temperature stress.

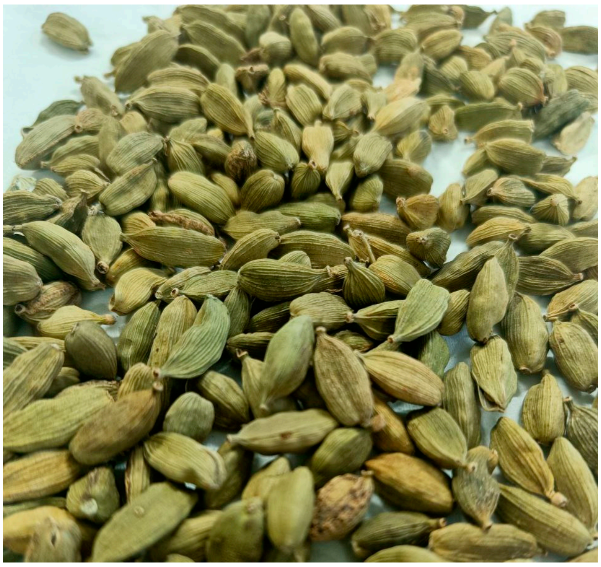

Figure S2. Dried fruits used to obtain the hydrodistillate EO.
